# Phytochemical and Biological Activities of *Euphorbia clavarioides* Boiss., a Traditional Wound-Healing Plant

**DOI:** 10.3390/plants15101550

**Published:** 2026-05-19

**Authors:** Khulile Ngcukana, Siphamandla Qhubekani Njabuliso Lamula, Thando Bhanisa, Sandisiwe Maposa, Anathi Dambuza, Martha Wium, Juliano Domiraci Paccez, Luiz Fernando Zerbini, Lisa V. Buwa-Komoreng

**Affiliations:** 1 Infectious Diseases and Medicinal Plants, Department of Biotechnology and Biological Sciences, Faculty of Science and Agriculture, University of Fort Hare, Private Bag X1314, Alice 5700, South Africa; 201716297@ufh.ac.za (K.N.); 201927865@ufh.ac.za (T.B.); smaposa@ufh.ac.za (S.M.); lbuwa@ufh.ac.za (L.V.B.-K.); 2 Department of Chemistry, Faculty of Science and Agriculture, University of Fort Hare, Private Bag X1314, Alice 5700, South Africa; 3 International Centre for Genetic Engineering and Biotechnology (ICGEB), Weirner & Beit Building, Anzio Rd, Observatory, Cape Town 7935, South Africajuliano.paccez@hc.fm.usp.br (J.D.P.); luiz.zerbini@icgeb.org (L.F.Z.)

**Keywords:** *Euphorbia clavarioides*, phytochemicals, LC-MS, antioxidant activity, anti-inflammatory activity, anticancer activity, FTIR

## Abstract

*Euphorbia clavarioides* Boiss. is traditionally used in wound healing and other medicinal applications. Its bioactive compounds and pharmacological potential remain underexplored. This study investigated the phytochemical composition, antioxidant, anti-inflammatory, and anticancer activities of *E. clavarioides* Boiss. traditionally used in wound healing. Plant extracts were characterized using phytochemical screening, Fourier-transform infrared spectroscopy (FTIR), and liquid chromatography–mass spectrometry (LC-MS). Antioxidant activity was evaluated via DPPH and nitric oxide (NO) scavenging assays, anti-inflammatory effects through nitrite inhibition in LPS-stimulated RAW 264.7 macrophages, and anticancer potential using the MTT assay against DU-145, PC-3, SKU-T, and AGS cell lines. Phytochemical screening confirmed tannins, phlobatannins, saponins, flavonoids, alkaloids, steroids, terpenoids, and cardiac glycosides. FTIR spectra of aqueous extracts revealed peaks at 2990.66 cm^−1^ (O–H), 1738.68 cm^−1^ (C=O), 1217.22 cm^−1^ (C–N), and 527.37 cm^−1^ (C–Cl). LC-MS profiling identified diverse metabolites, including phenolics (pseudolaroside B, cinnamtannin A2, (−)-medicarpin, butyrolactol A) and terpenoids (zerumbone, sclareol isomer, diterpenoid-like compounds), underpinning the plant’s bioactivity. Methanol extracts exhibited the strongest DPPH scavenging activity (IC_50_ = 755.71 µg/mL), whereas aqueous and ethanol extracts demonstrated superior NO scavenging. Ethanol extracts showed maximal anti-inflammatory activity, while aqueous extracts induced pro-inflammatory effects. Cytotoxicity assays indicated negligible toxicity. In anticancer assays, ethanol and methanol extracts significantly inhibited the proliferation of all tested cell lines at 100 µg/mL, exceeding drug control, whereas aqueous extracts displayed lower activity. The bioactive compounds in *E. clavarioides* support its traditional wound-healing use and demonstrate mechanistic antioxidant, anti-inflammatory, and anticancer activities, highlighting its potential as a source of multi-target natural therapeutics.

## 1. Introduction

Plants are known to be abundant in phytochemicals, which are bioactive substances with substantial medicinal benefits for humans [1]. These secondary metabolites contribute to plant characteristics such as colour, flavour, and aroma, while providing protection against environmental stressors, pathogens, and diseases [1]. Importantly, medicinal plants have been shown to exhibit therapeutic potential against a wide range of human diseases, often with relatively low toxicity in preclinical studies [2]. Common classes of bioactive compounds include alkaloids, tannins, phlobatannins, saponins, flavonoids, steroids, terpenoids, cardiac glycosides, and anthraquinones [3]. Phytochemicals have demonstrated therapeutic potential against diverse conditions, including diabetes, osteoporosis, gastrointestinal disorders, menopausal symptoms, neurological and immunological disorders, and cancer [4,5]. In particular, several phytochemicals exert anticancer activity by targeting dysregulated molecular pathways, modulating cell growth regulators, reducing oxidative stress, inhibiting angiogenesis, and inducing apoptosis in rapidly proliferating cancer cells [4,6]. Additional pharmacological effects include suppression of carcinogen formation, DNA protection, hormone regulation, and immune modulation, collectively reducing tumour growth and progression [6,7].

Wound healing is a complex physiological process involving hemostasis, inflammation, proliferation, and tissue remodeling [6]. Inflammation is a protective immune response that defends the body against infection, injury, and foreign substances [8,9]. However, chronic inflammation is closely associated with cancer initiation and progression, contributing to approximately 15% of global cancers [10,11]. Flavonoids, terpenoids, and alkaloids are key phytochemicals responsible for anti-inflammatory activity, highlighting their potential in preventing inflammation-driven carcinogenesis [8]. Oxidative stress is another critical factor in cancer development. Free radicals, including reactive oxygen and nitrogen species (ROS and RNS), are highly reactive molecules generated endogenously during metabolism or exogenously via environmental exposure. Excess ROS and RNS damage cellular macromolecules, leading to DNA mutations and tumour development [12,13]. Antioxidants neutralize these radicals, protecting cells from oxidative damage and contributing to cancer prevention and management [14,15]. Cancer is characterized by uncontrolled cell proliferation and tumour formation, which may remain localized or metastasize to distant tissues [16]. Dysregulation of normal cell division allows damaged cells to escape apoptosis and accumulate, forming malignant masses [17,18]. Although chemotherapy is widely used, its associated adverse effects can directly cause or lead to the development of wounds, primarily by impairing the body’s natural ability to heal, inhibiting cell division, and causing direct toxicity to healthy tissue. This necessitates the exploration of safer, natural alternatives. *Euphorbia clavarioides* Boiss. traditionally used for both anticancer and wound healing, has not been extensively studied for its phytochemical composition or the biological mechanisms that may underpin its ethnomedicinal use. Investigating its antioxidant and anti-inflammatory properties can provide insights into its role in modulating wound repair, while anticancer studies can reveal additional therapeutic potential related to its ability to regulate oxidative stress and inflammation. Therefore, this study aimed to perform a comprehensive evaluation of the phytochemical composition and biological activities of *E. clavarioides* plant extracts and explore its broader pharmacological potential.

## 2. Results

### 2.1. Extraction Yield

The extraction yields of *E. clavarioides* exhibited considerable variation based on the solvent employed (Table 1). The aqueous extract exhibited the highest yield (14.5%), followed by ethanol (2.8%), methanol (1.3%), and hexane (0.4%). This trend reflects the predominance of polar constituents within the plant matrix, which are more efficiently extracted using polar solvents such as water.

### 2.2. Qualitative Phytochemical Analysis

Using the qualitative phytochemical screening to detect phytochemicals in plant materials and extracts of *E. clavarioides*. The qualitative analysis revealed the presence of tannins, phlobatannins, saponins, flavonoids, alkaloids, steroids, terpenoids, and cardiac glycosides (Table 2). Flavonoids were strongly positive (++), whereas all other metabolites were positive (+).

### 2.3. Fourier Transform Infrared (FTIR) Spectroscopic Analysis

**Figure 1:**
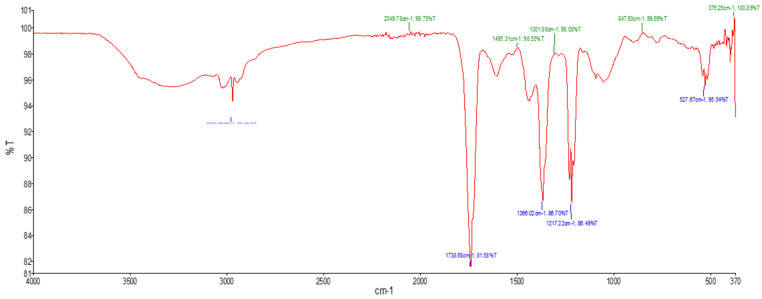
FTIR spectrum of *E. clavarioides* Boiss. aqueous extracts.

The FTIR was conducted to identify functional groups present in *E. clavarioides* in the aqueous extract. FTIR analysis was conducted on the aqueous extract as a representative sample, and it is the universal solvent that is commonly used by traditional healers. FTIR spectra of aqueous extract showed characteristic absorption bands at 2990.66 cm^−1^ (O–H stretch), 1738.68 cm^−1^ (C=O stretch), 1366.02 cm^−1^ (O–H stretch, alcoholic group), 1217.22 cm^−1^ (C–N stretch), and 527.37 cm^−1^ (C–Cl stretch). These bands corresponded to functional groups including carboxylic acids, carboxyl groups, phenols, amines, and alkyl halides, highlighting the presence of diverse phytocompounds (Figure 1; Table 3).

### 2.4. LC-MS Screening of Secondary Metabolites

**Figure 2:**
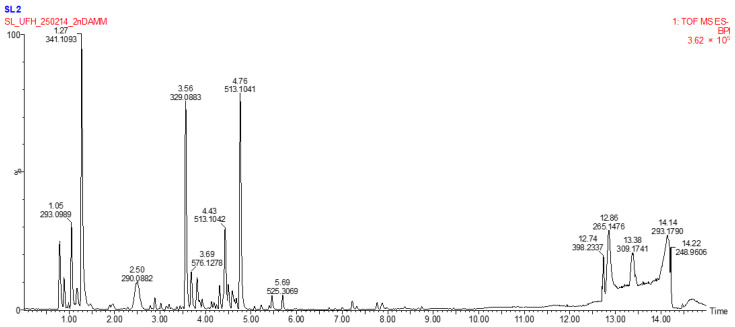
Basic Peak Chromatogram of methanolic extract of *E. clavarioides* Boiss.; negative ion mode.

**Figure 3:**
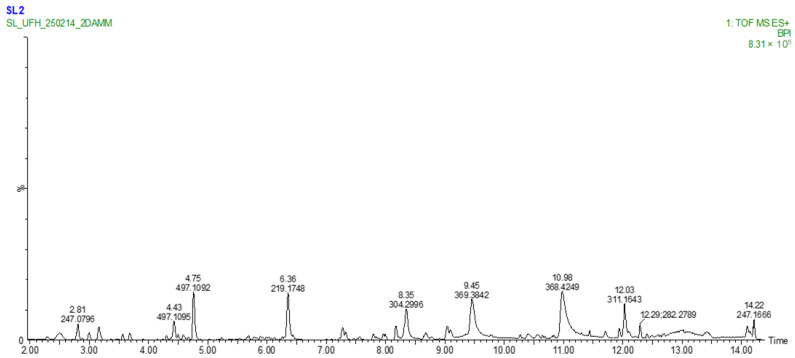
Basic Peak Chromatogram of methanolic extract of *E. clavarioides* Boiss.; positive ion mode.

The LC–MS analysis was conducted to provide representative metabolomic insight into the bioactive constituents of *E. clavarioides*. The methanol extract was chosen because it demonstrated the richest phytochemical profile and comparatively stronger biological activity in the initial assays. Non-targeted UHPLC-HRMS/MS metabolomic profiling of methanolic extracts of *E. clavarioides* revealed a diverse array of metabolites (Figure 2 and Figure 3; Table 4 and Table 5). Dual-mode analysis (negative and positive ionization) allowed detection of polar, mid-polar, and lipophilic metabolites.

#### 2.4.1. Negative Ion Mode

Negative ion mode analysis highlighted phenolic acids, flavonoids, tannins, and glycosylated metabolites, primarily eluting between 2.3 and 5.7 min. Key metabolites annotated at MSI confidence level 2 included Pseudolaroside B (*m*/*z* 329.08829, RT 3.56 min), which exhibited characteristic fragment ions at *m*/*z* 167.0341 and 149.0243, consistent with sequential sugar losses and a glycosidic phenolic structure. Cinnamtannin A2 (*m*/*z* 576.12775, RT 3.68 min) produced fragments at *m*/*z* 407.0812 and 289.0715, indicative of retro-Diels–Alder (RDA) and heterocyclic ring fissions typical of B-type proanthocyanidin dimers. Similarly, (−)-Medicarpin (*m*/*z* 269.08210, RT 4.44 min) showed fragments at *m*/*z* 254.0589 and 237.0492, confirming its pterocarpan skeleton. (confidence level 3).

#### 2.4.2. Positive Ion Mode

Positive ion mode preferentially detected terpenoids and lipophilic metabolites, eluting between 7.0 and 12.1 min. Notable compounds included Zerumbone (*m*/*z* 219.1746, RT 7.27 min) generated ions at *m*/*z* 201.1635 and 147.1012, reflecting cleavage of the α,β-unsaturated ketone moiety, while Sclareol isomer (*m*/*z* 309.2787, RT 12.09 min) yielded fragments at *m*/*z* 291.2678 and 273.2571, corresponding to sequential losses of water and methanol, consistent with a diterpenoid alcohol structure (confidence level 3).

### 2.5. Antioxidant Activity

The antioxidant potential of *E. clavarioides* extracts was evaluated using DPPH and nitric oxide (NO) radical scavenging assays. These complementary assays provide insight into the ability of the extracts to neutralize stable free radicals and reactive nitrogen species, respectively.

#### 2.5.1. DPPH Radical Scavenging

All extracts of *E. clavarioides* demonstrated concentration-dependent DPPH radical scavenging activity, although marked differences were observed among extraction solvents (Table 6). The methanol extract exhibited the strongest antioxidant activity, with an IC_50_ value of 755.71 ± 7.26 µg/mL, indicating a higher hydrogen-donating capacity relative to the ethanol (IC_50_ = 1913.64 ± 3.78 µg/mL) and aqueous extracts (IC_50_ = 1952.50 ± 4.85 µg/mL). Ascorbic acid, used as a positive control, displayed significantly higher antioxidant activity (IC_50_ = 373.33 ± 2.82 µg/mL) than all plant extracts, as expected for a purified reference antioxidant.

#### 2.5.2. Nitric Oxide Scavenging

In contrast to the DPPH assay, aqueous extracts demonstrated the highest nitric oxide scavenging activity, with an IC_50_ value of 2668.89 ± 5.98 µg/mL, followed by ethanol (IC_50_ = 3061.54 ± 9.39 µg/mL) and methanol extracts (IC_50_ = 3663.33 ± 4.94 µg/mL) (Table 6). This suggests that polar compounds present in the aqueous extract may play a more prominent role in neutralizing reactive nitrogen species. Despite this activity, all extracts were less effective than ascorbic acid (IC_50_ = 723.53 ± 7.04 µg/mL).

### 2.6. Anti-Inflammatory Activity

**Figure 4:**
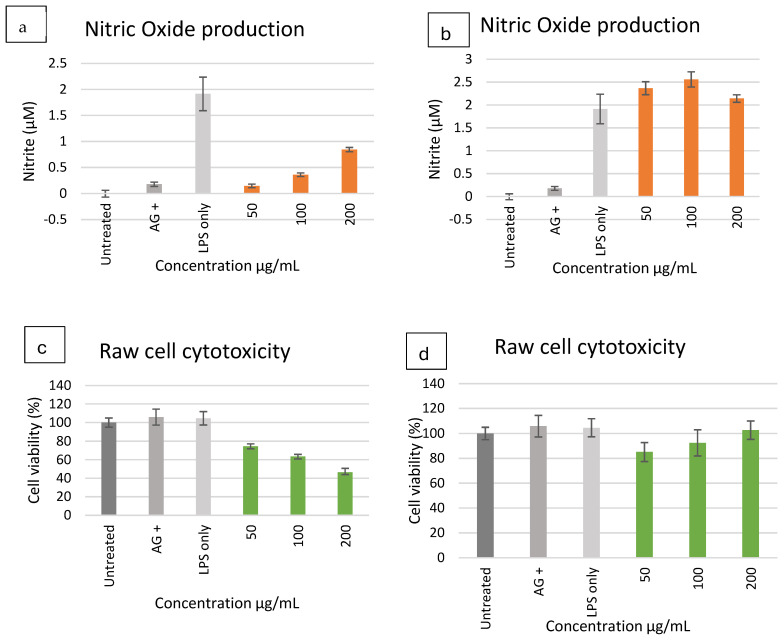
Nitric oxide production in LPS-activated macrophages (**a**,**b**) and cell viability (**c**,**d**) of cells treated with ethanol and water extracts. The bar graph represents quadruplicate values of one experiment. Error bars represent the standard deviation of the mean.

The effects of *E. clavarioides* ethanol and aqueous extracts on nitric oxide (NO) production and cell viability in LPS-stimulated RAW 264.7 macrophages are shown in Figure 4. Ethanol extracts significantly reduced NO production compared to the LPS control at concentrations ≤50 µg/mL (Figure 4a). The greatest reduction in nitrite levels was observed at 50 µg/mL, where values were comparable to those obtained with the positive control, aminoguanidine. At concentrations above 50 µg/mL, the inhibitory effect on NO production decreased. Aqueous extracts maintained high cell viability across all tested concentrations (Figure 4d), with cell viability at 200 µg/mL exceeding that of LPS-treated cells. No cytotoxic effects were observed. However, aqueous extracts significantly increased NO production relative to LPS-stimulated cells at all concentrations tested (Figure 4b), with the highest nitrite levels recorded at 100 µg/mL.

### 2.7. Anticancer Activity

**Figure 5:**
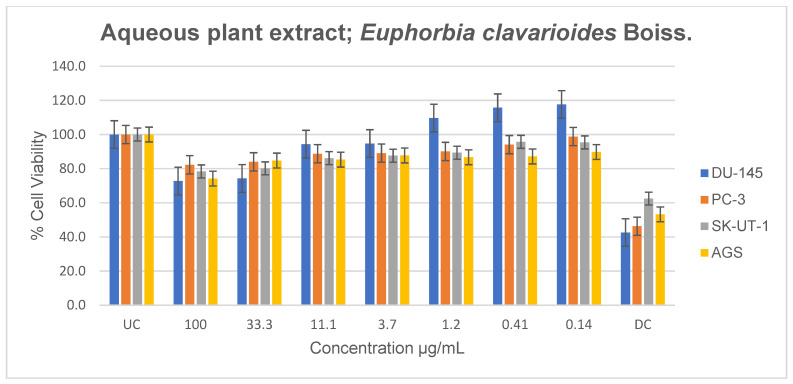
Antiproliferative effects of *E. clavarioides* aqueous extract against selected cancer cell lines (UC and DC mean positive control and drug control, respectively). The bars illustrate the cell viability of the different cancer cell lines at various concentrations. Means are an average of six concentrations for each extract ± SD.

**Figure 6:**
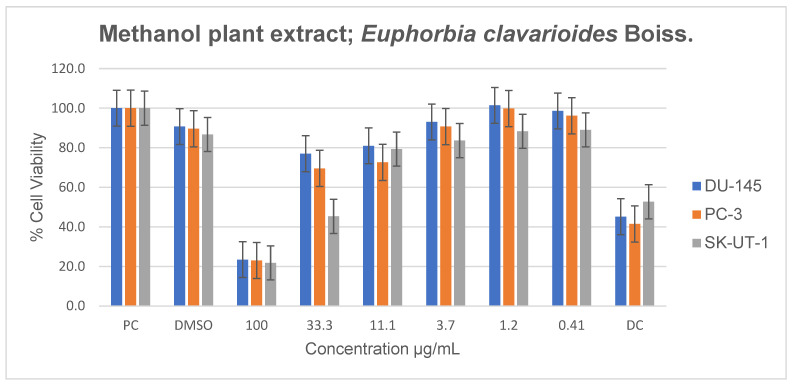
Antiproliferative effects of *E. clavarioides* methanol extract against selected cancer cell lines (UC and DC mean positive control and drug control, respectively). The bars illustrate the cell viability of the different cancer cell lines at various concentrations. Means are an average of six concentrations for each extract ± SD.

**Figure 7:**
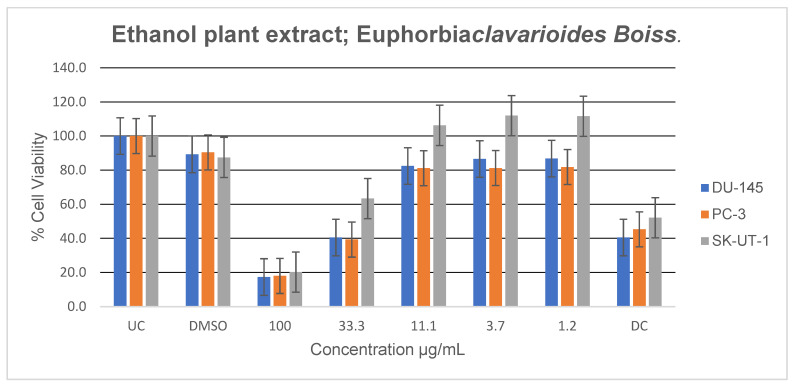
Antiproliferative effects of *E. clavarioides* ethanol extract against selected cancer cell lines (UC and DC mean positive control and drug control, respectively). The bars illustrate the cell viability of the different cancer cell lines at various concentrations. Means are an average of six concentrations for each extract ± SD.

The antiproliferative effects of *E. clavarioides* aqueous, methanolic, and ethanolic extracts on DU-145, PC-3, SK-UT-1, and AGS cancer cell lines were evaluated using the MTT assay (Figure 5, Figure 6 and Figure 7). Docetaxel (Taxotere) served as the drug control, while untreated cells were used as the negative control. Aqueous extracts exhibited low antiproliferative activity across all tested cell lines compared to the untreated control (Figure 5). In DU-145 cells, reduced proliferation was observed at concentrations of 33.3 and 100 µg/mL. Although lower concentrations (0.14–1.2 µg/mL) showed a slight increase in proliferation relative to the untreated control, this effect was not statistically significant. Moderate, dose-dependent antiproliferative effects were observed in PC-3, SK-UT-1, and AGS cell lines.

Methanolic extracts significantly reduced cell viability in all cancer cell lines at 100 µg/mL, with noticeable antiproliferative effects when compared to those of the untreated control in SK-UT-1 cells (Figure 6). Reduced proliferation was observed in DU-145 cells at all concentrations except 1.2 µg/mL. In PC-3 cells, antiproliferative effects were most pronounced between 11.1 and 100 µg/mL. A dose-dependent decrease in cell viability was also observed in SK-UT-1 cells.

Ethanolic extracts demonstrated the strongest antiproliferative activity among all extracts (Figure 7). Significant inhibition of cell proliferation was observed across all cancer cell lines at higher concentrations, with stronger activity when compared to that of the untreated control. In SK-UT-1 cells, increased proliferation was noted at lower concentrations (1.2–11.1 µg/mL); however, this increase was not statistically significant. Marked antiproliferative effects were observed at 33.3 and 100 µg/mL.

## 3. Discussion

### 3.1. Extraction Yield

Notwithstanding the comparatively low yield achieved with methanol, this extract exhibited a more complex metabolomic profile and enhanced biological activity, especially in antioxidant and anticancer evaluations. UHPLC-HRMS/MS analysis revealed that the methanol extract contained diverse bioactive metabolites, including phenolic compounds such as cinnamtannin A2 and (−)-medicarpin, as well as terpenoid constituents identified in positive ion mode. These compounds are widely associated with radical scavenging, anti-inflammatory modulation, and cytotoxic effects. The disparity between extraction yield and biological activity highlights that extraction efficiency does not necessarily correlate with bioactive compound abundance. While aqueous extraction recovered a larger mass of polar constituents, these may include inactive or less potent compounds. In contrast, methanol appears to selectively extract structurally diverse and pharmacologically active metabolites, which likely contribute to the observed antioxidant, anti-inflammatory, and antiproliferative effects. These results support the use of methanol for metabolomic profiling and bioactivity-guided studies of *E. clavarioides* and highlight the significance of solvent selection in phytochemical investigations.

### 3.2. Phytochemical Composition

Qualitative phytochemical screening of *E. clavarioides* extracts confirmed the presence of tannins, phlobatannins, saponins, flavonoids, steroids, terpenoids, and cardiac glycosides. These classes of secondary metabolites are widely associated with diverse pharmacological activities, including antioxidant, anti-inflammatory, and anticancer effects [4,6,7]. The detection of tannins aligns with previous reports in other Euphorbia species [20]. Tannins are well recognized for their antioxidant and antimicrobial properties, as well as their role in wound healing and anti-inflammatory responses [21]. Phlobatannins were also identified; although not previously reported in *E. clavarioides*, they have been documented in other medicinal plants and are associated with antioxidant, analgesic, and wound-healing activities [22]. The presence of saponins and flavonoids corroborates earlier findings in Euphorbia species [20,23]. These compounds are known for their broad biological activities, including immunomodulatory, anti-inflammatory, and anticancer properties [24,25]. Similarly, steroids and terpenoids are major classes of plant secondary metabolites and have been widely reported to exhibit anti-inflammatory, antioxidant, and cytotoxic activities [26,27,28]. Cardiac glycosides were also detected, supporting earlier reports in *E. clavarioides* [23]. Beyond their established role in cardiovascular therapy [29], these compounds have demonstrated pro-apoptotic effects in certain cancer cell lines [30]. Although alkaloids were not detected in the present study, previous investigations have reported their occurrence in this species [23], suggesting possible variability due to extraction methods or plant chemotype.

### 3.3. Fourier Transform Infrared Spectroscopy Analysis

The FTIR spectra of *E. clavarioides* extracts revealed characteristic absorption bands corresponding to major functional groups. Prominent peaks were assigned to O–H stretching, C=O stretching, C–N stretching, and C–Cl stretching vibrations. These bands indicate the presence of functional groups associated with carboxylic acids, phenolic compounds, amines, and alkyl halides. The broad O–H stretching bands suggest the occurrence of hydroxyl-containing compounds, including polyphenols and flavonoids. The C=O stretching bands are consistent with carbonyl-containing compounds, including flavonoids and carboxylic acids. The presence of C–N stretching bands indicates amine-containing compounds, while C–Cl stretching bands correspond to alkyl halides. These findings are comparable with previous FTIR reports for Euphorbia species, where similar functional groups were detected [31,32,33,34]. FTIR analysis was used primarily as a supportive technique to identify functional groups present in the extracts rather than to identify individual compounds, while LC–MS served as the principal analytical method for metabolite identification.

### 3.4. LC-MS Screening of Secondary Metabolites

Non-targeted UHPLC-HRMS/MS metabolomic profiling of the methanolic extract of *E. clavarioides* revealed a chemically diverse metabolome, as illustrated in the basic peak chromatograms (Figure 2 and Figure 3) and summarized in Table 3 and Table 4. Dual ionization (negative and positive modes) enabled broad metabolite coverage, facilitating detection of polar, mid-polar, and lipophilic constituents across a wide retention time (RT) range. High mass accuracy (Δ ppm < 2 for most annotated compounds) supported tentative identification at Metabolomics Standards Initiative (MSI) confidence levels 2 and 3. The detected metabolite classes align with those previously reported in biologically active Euphorbia species [20,35,36]. The methanol extract was selected for LC–MS metabolomic profiling because methanol is widely recognized as an efficient solvent for extracting a broad range of secondary metabolites, including phenolics, flavonoids, and terpenoids. These compounds are frequently associated with antioxidant and anti-inflammatory activities. Therefore, LC–MS analysis of the methanol extract was conducted to obtain a representative chemical profile of the bioactive constituents present in *E. clavarioides*.

#### 3.4.1. Negative Ion Mode

Negative ion mode predominantly detected polar metabolites eluting between 2.3 and 5.7 min, characterized by phenolic acids, flavonoids, tannins, and glycosylated derivatives. MSI level 2 annotations included pseudolaroside B, cinnamtannin A2, and (−)-medicarpin, identified with low mass errors (≤1.61 ppm). These polyphenolic and pterocarpan-type compounds are widely associated with antioxidant and anti-inflammatory activities through free radical scavenging, metal chelation, and modulation of redox-sensitive signaling pathways such as NF-κB. Proanthocyanidin-type compounds like cinnamtannin A2 have also demonstrated antiproliferative effects in various cancer models. The predominance of phenolic metabolites in this ion mode therefore provides chemical support for the observed DPPH and nitric oxide scavenging activities.

#### 3.4.2. Positive Ion Mode

Positive ion mode preferentially detected terpenoids and other lipophilic metabolites, primarily eluting between 7.0 and 12.1 min. Zerumbone and a sclareol isomer were annotated at MSI level 2 with minimal mass deviation (≤1.28 ppm). Terpenoids such as these are well documented for anti-inflammatory and anticancer activities, including inhibition of nitric oxide production, suppression of NF-κB signaling, induction of apoptosis, and cell cycle arrest. Additional diterpenoid and triterpenoid-like features (MSI level 3) further reflect the terpenoid-rich nature of the extract, a chemotaxonomic characteristic of the genus Euphorbia. Collectively, the LC–MS profile reveals a synergistic framework of phenolic antioxidants and bioactive terpenoids. This chemical composition provides mechanistic insight into the antioxidant, anti-inflammatory, and antiproliferative activities observed in *E. clavarioides,* reinforcing its pharmacological potential.

### 3.5. Antioxidant Activity

The antioxidant potential of *E. clavarioides* extracts was evaluated using DPPH and nitric oxide (NO) radical scavenging assays (Table 5). In the DPPH assay, the standard antioxidant ascorbic acid exhibited the strongest activity with an IC_50_ value of 373.33 µg/mL. Among the plant extracts, the methanol extract demonstrated the highest radical-scavenging activity (IC_50_ = 755.71 µg/mL), followed by the ethanol extract (IC_50_ = 1913.64 µg/mL) and the aqueous extract (IC_50_ = 1952.50 µg/mL). These findings are consistent with those reported by Majid et al. [37], who observed stronger antioxidant activity for ascorbic acid compared with plant extracts. However, the present results differ from those of Mbhele et al. [38], who reported the ethanol extract as exhibiting the strongest antioxidant activity. In the nitric oxide scavenging assay, ascorbic acid again showed the highest activity (IC_50_ = 723.53 µg/mL). Among the plant extracts, the aqueous extract exhibited the strongest NO radical scavenging activity (IC_50_ = 2668.89 µg/mL), followed by the ethanol extract (IC_50_ = 3061.54 µg/mL) and the methanol extract (IC_50_ = 3663.33 µg/mL). The comparatively higher activity of the aqueous extract contrasts with previous studies that reported stronger activity for ethanol extracts [38]. The antioxidant activity observed in this study may be attributed to the presence of bioactive phytochemicals identified through qualitative phytochemical screening and LC–MS profiling, including flavonoids, tannins, saponins, steroids, and terpenoids. These compounds are well known for their ability to neutralize reactive oxygen species (ROS) and reactive nitrogen species through hydrogen donation, electron transfer, and metal-chelating mechanisms [27,28,35,39]. Oxidative stress plays a critical role in the pathogenesis of several chronic diseases, including cancer and inflammatory disorders. Excessive ROS can damage cellular macromolecules such as DNA, lipids, and proteins, ultimately leading to cellular dysfunction and tumorigenesis. The antioxidant activity observed in the present study, particularly in the methanol extract, suggests that *E. clavarioides* contain bioactive compounds capable of scavenging free radicals and mitigating oxidative stress. Notably, phenolic compounds tentatively identified by LC–MS analysis, such as cinnamtannin A2 and (−)-medicarpin, are reported to possess strong radical-scavenging properties due to their hydroxyl groups, which can donate hydrogen atoms to neutralize free radicals. These findings are consistent with previous reports describing strong antioxidant activity in other Euphorbia species, including *E. parviflora* [35], *E. milii*, *E. trigona*, and *E. antiquorum* [40]. Collectively, these results support the antioxidant potential of *E. clavarioides* and highlight its potential as a source of natural compounds capable of protecting cells against oxidative damage. Moreover, the antioxidant activity observed in *E. clavarioides* extracts may also have important implications for inflammatory processes. Oxidative stress and inflammation are closely interconnected biological phenomena, where excessive production of reactive oxygen species can activate pro-inflammatory signaling pathways such as NF-κB and MAPK. These pathways regulate the expression of inflammatory mediators, including nitric oxide (NO), cytokines, and prostaglandins. Therefore, the ability of *E. clavarioides* extracts to scavenge free radicals may contribute to the modulation of inflammatory responses, prompting further evaluation of their anti-inflammatory potential.

### 3.6. Anti-Inflammatory Assay

The anti-inflammatory potential of *E. clavarioides* extracts was evaluated using lipopolysaccharide (LPS)-stimulated RAW 264.7 macrophages. Inflammation is closely associated with oxidative stress and cancer development. Chronic inflammatory responses contribute to tumor initiation and progression through the activation of signaling pathways such as nuclear factor kappa B (NF-κB) and mitogen-activated protein kinase (MAPK). These pathways regulate the production of pro-inflammatory mediators, including nitric oxide (NO), cytokines, and prostaglandins. In the present study, the ethanol extract significantly inhibited nitric oxide production in LPS-stimulated RAW 264.7 macrophages, indicating notable anti-inflammatory activity. This effect may be associated with the presence of terpenoid compounds identified through LC–MS analysis, including zerumbone and sclareol derivatives. Previous studies have demonstrated that zerumbone suppresses inflammatory responses by inhibiting NF-κB activation and downregulating inducible nitric oxide synthase (iNOS) expression, thereby reducing NO production. The observed decrease in NO levels in this study may therefore involve similar molecular mechanisms. Ethanol extracts exhibited strong anti-inflammatory activity at lower concentrations but showed increased cytotoxicity at higher concentrations, which may have influenced NO inhibition measurements (Figure 4). This cytotoxicity is consistent with previous reports indicating that species within the Euphorbia genus contain biologically active diterpenoids and other compounds with cytotoxic properties [36,41]. In contrast, the aqueous extract maintained high cell viability across all tested concentrations but induced elevated nitrite production, suggesting a pro-inflammatory response. This observation may be related to the physiological role of inflammation during the early stages of wound healing, which aligns with the traditional use of *E. clavarioides* in the treatment of wounds [38,42]. Controlled inflammatory responses are essential for initiating tissue repair processes, including immune cell recruitment and removal of damaged tissue. These findings are partially consistent with previous studies on Euphorbia species, which have reported anti-inflammatory activities mediated by latex-derived metabolites, flavonoids, and polyphenols [43,44,45]. However, the pro-inflammatory activity observed in the aqueous extract contrasts with the findings of Mbhele et al. [38], who reported anti-inflammatory effects through inhibition of enzymes such as xanthine oxidase, 15-lipoxygenase, and cyclooxygenase-2. Nevertheless, the ethanol extract of *E. clavarioides* demonstrates promising anti-inflammatory potential, while the activity of the aqueous extract may reflect the plant’s traditional wound-healing role, where controlled inflammation contributes to the early stages of tissue regeneration. Beyond inflammation control, the inhibition of nitric oxide production and modulation of inflammatory signaling pathways may also influence cancer development. Chronic inflammation is widely recognized as a key contributor to tumor initiation and progression, as inflammatory mediators promote cell proliferation, angiogenesis, and metastasis. Consequently, natural compounds capable of regulating inflammatory responses may also exhibit anticancer potential. Given the antioxidant and anti-inflammatory properties observed in *E. clavarioides* extracts, the antiproliferative effects of the plant were further investigated against selected human cancer cell lines.

### 3.7. Anticancer Activity

Medicinal plants exert anticancer effects largely through interconnected antioxidant and anti-inflammatory mechanisms. Chronic inflammation and oxidative stress are closely linked drivers of carcinogenesis. Excessive production of reactive oxygen species (ROS) and reactive nitrogen species (RNS), including nitric oxide (NO), promotes DNA damage, genomic instability, and activation of pro-survival signaling pathways such as nuclear factor kappa B (NF-κB) and mitogen-activated protein kinase (MAPK), thereby facilitating tumour initiation and progression [46,47]. Persistent macrophage activation and inducible nitric oxide synthase (iNOS)-mediated NO overproduction further contribute to a pro-tumorigenic microenvironment [48,49]. In the present study, *E. clavarioides* extracts demonstrated measurable antioxidant activity in both DPPH and nitric oxide scavenging assays, together with notable antiproliferative effects in the methanol and ethanol extracts. The strong DPPH radical scavenging activity observed in the methanol extract indicates effective hydrogen-donating and electron-transfer capacity, which is typically associated with phenolic and flavonoid compounds. These compounds are known to mitigate oxidative DNA damage and regulate redox-sensitive transcription factors, thereby suppressing cancer cell proliferation [50]. Although nitric oxide inhibition in the ethanol extract could not be conclusively interpreted due to cytotoxic effects observed in RAW 264.7 macrophages, this cytotoxicity may itself contribute to anticancer activity. Terpenoids, particularly diterpenoids detected in the LC–MS analysis, are widely reported to induce apoptosis through mitochondrial pathways, activation of caspases, and cell-cycle arrest [36]. The presence of terpenoid compounds such as zerumbone and sclareol derivatives further supports this mechanism, as these metabolites have been reported to suppress NF-κB signaling and promote apoptosis in various cancer models. The coexistence of polyphenolic compounds (antioxidant modulators) and terpenoids (pro-apoptotic and cytotoxic agents) suggest a dual mechanism of action. Firstly, phenolic compounds may restore redox balance and suppress inflammation-related signaling pathways. Secondly, terpenoid constituents may directly induce cancer cell death through cytotoxic and apoptosis-inducing mechanisms. Therefore, the anticancer activity observed in *E. clavarioides* extracts may be associated with their antioxidant and immunomodulatory properties; however, direct mechanistic links were not established in the present study and require further investigation. Nonetheless, by reducing oxidative stress, modulating nitric oxide-mediated inflammatory pathways, and inducing cytotoxic effects in tumour cells, the extracts may disrupt multiple processes involved in cancer progression. These findings highlight the therapeutic potential of *E. clavarioides* as a source of multi-target bioactive phytochemicals and warrant further studies focusing on bioactivity-guided isolation and molecular characterization of the active compounds.

## 4. Methods

### 4.1. Collection of Plant Material

*Euphorbia clavarioides* Boiss. was collected between the 23rd and 30th of September 2023 in Pietermaritzburg, KwaZulu-Natal Province, South Africa. Additional plant material was obtained from personnel working in nature conservation and the Durban herbal market in KwaZulu-Natal. Proper identification of the species was completed by a qualified taxonomist, and a voucher specimen (BUW011SNGC01) was deposited at the herbarium of the Faculty of Science and Agriculture, University of Fort Hare, South Africa.

### 4.2. Preparation of Extracts

Collected plant material was washed under running tap water to remove soil residues and chopped into small pieces. The whole plant material was air-dried at room temperature and ground into fine powder using a blender. Four portions of 30 g each were extracted separately with 300 mL of distilled water, 80% ethanol (*v*/*v*), methanol, and hexane, and placed on a shaker for 24 h at room temperature. The extracts were filtered through Whatman No. 1 filter paper. The aqueous extract was evaporated to dryness using a freeze-dryer, while the ethanol, methanol, and hexane extracts were concentrated using a rotary evaporator. The dried extracts were stored at 4 °C until further analysis. The selection of solvents with different polarities was intended to maximize the extraction of diverse phytochemical classes. Polar solvents such as water and ethanol facilitate the extraction of hydrophilic compounds, including flavonoids and glycosides, whereas methanol efficiently extracts phenolic compounds. Hexane, a nonpolar solvent, enables the extraction of lipophilic metabolites such as terpenoids.

### 4.3. Qualitative Phytochemical Analysis

Qualitative phytochemical analysis of alkaloids, tannins, phlobotanins, saponins, flavonoids, steroids, terpenoids and cardiac glycosides was performed according to the standard procedures described by Harborne [51], Trease and Evans [52], Sofowora [53], and Edeoga et al. [54].

### 4.4. Fourier Transform Infrared Spectroscopy Analysis

The FTIR analysis was performed to identify the functional groups present in the plant extracts. Approximately 10 mg of crude aqueous extract was mixed with 100 mg of KBr to form a translucent pellet. The pellet was analyzed using a Perkin Elmer Spectrum 100 FTIR spectrometer (Shelton, CT, USA). The scan range was set from 400 to 4000 cm^−1^ with a resolution of 4 cm^−1^.

### 4.5. LC-MS Screening for Secondary Metabolites

The plant material was first washed, freeze-dried using a Christ Alpha 1-4 LDplus freeze dryer ((Christ Alpha 1-4 LDplus, Martin Christ Gefriertrocknungsanlagen GmbH, Osterode am Harz, Germany)), and then ground into a fine homogeneous powder using a mechanical grinder. Exactly 1.0 g of the powdered sample was subjected to ultrasonication-assisted extraction using a Branson 3800 ultrasonic cleaner ((Branson 3800 Ultrasonic Cleaner, Danbury, CT, USA)) with 20 mL of 80% aqueous methanol (*v*/*v*) for 30 min at room temperature. The resulting extract was centrifuged at 10,000× *g* for 10 min using an Eppendorf 5430 R centrifuge (Eppendorf 5430 R, Hamburg, Germany), and the supernatant was collected. The extraction procedure was repeated twice to ensure maximum metabolite recovery. The pooled supernatants were then filtered through a 0.22 µm PTFE syringe filter (Millipore, Burlington, MA, USA) and transferred into LC–MS vials for subsequent analysis.

UHPLC–HRMS/MS Analysis Chromatographic separation was carried out using a Vanquish Horizon UHPLC system (Thermo Fisher Scientific, Dreieich, Germany) fitted with a reversed-phase ACQUITY UPLC^®^ HSS T3 column (100 mm × 2.1 mm, 1.8 µm; Waters, Ireland) maintained at 40 °C. The mobile phase consisted of 0.1% formic acid in water (solvent A) and 0.1% formic acid in acetonitrile (solvent B). The gradient elution program was set as follows: 0–2 min, 5% B; 2–15 min, 5–95% B; 15–17 min, 95% B; 17–17.1 min, 95–5% B; and 17.1–20 min, 5% B to allow column re-equilibration. The flow rate was maintained at 0.4 mL/min, and the injection volume was 2 µL. High-resolution mass spectrometric detection was performed using a Q-Exactive™ Plus Hybrid Quadrupole-Orbitrap™ mass spectrometer (Thermo Fisher Scientific, Germany) equipped with a Heated Electrospray Ionization (H-ESI II) source. Data acquisition was conducted in negative ionization mode. Full-scan mass spectra were recorded over an *m*/*z* range of 100–1500 at a resolution of 70,000, with an automatic gain control (AGC) target of 3 × 10^6^ and a maximum injection time of 100 ms. The five most intense precursor ions were selected for fragmentation using Higher-Energy Collisional Dissociation (HCD) with stepped normalized collision energies of 20, 40, and 60 eV. MS/MS spectra were acquired at a resolution of 17,500, and dynamic exclusion was set to 15 s to avoid repeated fragmentation of the same ions. The acquired raw data were processed using Compound Discoverer™ 3.3 software, which included retention time alignment, peak detection (minimum intensity 500,000; signal-to-noise ratio ≥ 3), gap filling, adduct and isotope grouping, and background subtraction using procedural blanks. Metabolite annotation was performed by comparison against multiple databases, including HMDB, mzCloud™, PubChem, ChemSpider, and COCONUT. Compound identification confidence levels were assigned according to the Metabolomics Standards Initiative (MSI) guidelines.

### 4.6. Antioxidant Assay

The antioxidant activity of the extracts was evaluated using DPPH radical scavenging and nitric oxide (NO) scavenging assays. All experiments were performed in triplicate.

#### 4.6.1. DPPH Radical Scavenging Activity

The DPPH radical scavenging activity of *E. clavarioides* extracts was evaluated according to the method described by Dambuza et al. [55], with slight modifications. A 0.135 mM solution of 2,2-diphenyl-1-picrylhydrazyl (DPPH) was freshly prepared in methanol and protected from light to prevent degradation. Briefly, 1 mL of the DPPH solution was mixed with 0.1 mL of plant extract at varying concentrations (312.5, 625, 1250, and 2500 µg/mL). Ascorbic acid, prepared in methanol at similar concentrations, was used as the positive control, while methanol served as the negative control. A blank containing methanol and extract without DPPH was also prepared to correct for background absorbance. The reaction mixtures were vortexed thoroughly and incubated in the dark at room temperature (25 °C) for 30 min to allow complete interaction between the DPPH radicals and antioxidant compounds present in the extracts. After incubation, the decrease in absorbance was measured at 517 nm using a spectrophotometer. All experiments were conducted in triplicate, and results were expressed as mean ± standard deviation (SD).DPPH scavenging activity (%) was calculated as:

(1) $$\%\;DPPH\;scaveging\;activity=\frac{Absorbance\;of\;sample-Absorbance\;of\;blank}{Absornace\;of\;control-Absorbance\;of\;Blank}\times 100$$

#### 4.6.2. Nitric Oxide Scavenging Activity

Nitric oxide scavenging activity was determined according to the method described by Dambuza et al. [56]. Briefly, different concentrations of the extracts (312.5, 625, 1250, and 2500 µg/mL) were mixed with 2 mL of 10 mM sodium nitroprusside prepared in 0.5 mM phosphate-buffered saline (pH 7.4) and incubated at 25 °C for 180 min under light to facilitate NO generation. After incubation, 1 mL of the reaction mixture was combined with 1 mL of freshly prepared Griess reagent (equal volumes of 0.33% sulfanilic acid in 20% glacial acetic acid and 0.1% *N*-(1-naphthyl) ethylenediamine dihydrochloride in distilled water). The mixture was incubated for 30 min at room temperature in the dark. Absorbance was measured at 540 nm using a spectrophotometer. All experiments were performed in triplicate.

(2) $$NO\;radical\;scaveging\;activity\left(\%\right)=\frac{Absorbance\;of\;sample-Absorbance\;of\;blank}{Absorbance\;of\;control-Absorbance\;of\;Blank}\times 100$$

### 4.7. Anti-Inflammatory Activity

The anti-inflammatory activity of *E. clavarioides* extracts was evaluated using RAW 264.7 murine macrophage cells (Cellonex, South Africa), a well-established model for assessing modulation of inflammatory responses in vitro. Cells were maintained in Roswell Park Memorial Institute (RPMI-1640) medium supplemented with 10% fetal bovine serum (FBS), 1 mM L-glutamine, and 100 U/mL penicillin/100 µg/mL streptomycin and incubated at 37 °C in a humidified atmosphere containing 5% CO_2_ [57].

For the assay, cells were seeded into 96-well plates at a density of 1 × 10^5^ cells per well and allowed to adhere overnight. The following day, cells were treated with different concentrations of plant extracts (62.5, 125, and 250 µg/mL) in the presence of lipopolysaccharide (LPS, 500 ng/mL) to stimulate an inflammatory response. Aminoguanidine (AG) at 25, 50, and 100 µM served as a positive control for nitric oxide (NO) inhibition. After 24 h of incubation, the culture medium was collected, and nitrite levels, a stable metabolite of NO, were quantified using the Griess reaction. Briefly, equal volumes of sulfanilamide solution and *N*-(1-naphthyl) ethylenediamine dihydrochloride (NED) solution were added to the culture supernatant, incubated in the dark at room temperature, and the absorbance was measured at 540 nm using a microplate spectrophotometer. Nitrite concentrations were determined using a sodium nitrite standard curve, and results were expressed as % NO inhibition.

To evaluate cytotoxicity and confirm that NO inhibition was not due to reduced cell viability, an MTT assay was performed in parallel. After treatment, cells were incubated with MTT solution (0.5 mg/mL) for 30 min at 37 °C. The resulting formazan crystals were solubilized with DMSO, and absorbance was measured at 540 nm. Cell viability was calculated relative to untreated controls to ensure that observed anti-inflammatory effects were independent of cytotoxicity [58].

### 4.8. Anticancer Activity

The anticancer potential of *E. clavarioides* extracts was assessed using human cancer cell lines, including prostate carcinoma (DU-145 and PC-3), uterine leiomyosarcoma (SK-UT-1), and gastric adenocarcinoma (AGS), obtained from the American Type Culture Collection (ATCC). All cell lines were cultured in Dulbecco’s Modified Eagle Medium (DMEM) (ICGEB, Cape Town, South Africa) supplemented with 10% fetal bovine serum (FBS), 1 mM L-glutamine, 100 U/mL penicillin, and 100 µg/mL streptomycin. The cells were maintained at 37 °C in a humidified incubator with 5% CO_2_ (Thermo Fisher Scientific, Frederick, MD, USA). The antiproliferative activity of the plant extracts was evaluated in vitro using a modified MTT (3-(4,5-dimethylthiazol-2-yl)-2,5-diphenyltetrazolium bromide) assay, following the method described by Mosmann [59]. Cells were first trypsinized, counted using a hemocytometer, and seeded into 96-well plates at a density of 6 × 10^4^ cells per well in 100 µL of complete DMEM. The cells were allowed to attach and stabilize for 24 h prior to treatment. The methanol and ethanol extracts were dissolved in dimethyl sulfoxide (DMSO), whilst the aqueous extracts were dissolved in distilled water, and, subsequently, all the extracts were diluted in culture medium to obtain final concentrations ranging from 10 to 200 µg/mL. Each treatment was applied in triplicate wells, with untreated cells serving as the negative control, while wells containing only culture medium were used as blanks. The cells were then incubated with the extracts for 72 h under standard culture conditions. After incubation, 10 µL of MTT solution (2.5 mg/mL) was added to each well, and the plates were incubated for 4 h to allow metabolically active cells to convert the MTT reagent into formazan crystals. Subsequently, the culture medium was carefully removed, and 100 µL of 10% SDS in 0.1 N HCl was added to each well to dissolve the formazan crystals overnight. The absorbance was measured using a Thermo Multiskan Go microplate reader (Thermo Fisher Scientific, Waltham, MA, USA) at a wavelength of 595 nm after 72 h of treatment. The average absorbance value of untreated control wells was considered 100% cell viability, and the percentage of viable cells following treatment was calculated relative to this control.

Cell viability (%) was calculated using the following equation:

(3) $$Percentage\;cell\;viability=\frac{Absorbance\;of\;sample}{Absorbance\;of\;control}\times 100\%.$$

### 4.9. Statistical Analysis

All data processing, graphing, and organization were performed using Microsoft Excel 2013. Experiments were conducted in triplicate to ensure reproducibility. Statistical differences between samples were evaluated using one-way analysis of variance (ANOVA), and differences were considered statistically significant at *p* < 0.05. Results are presented as mean ± standard deviation (SD), and multiple comparisons among groups were performed using Duncan’s multiple range test.

## 5. Conclusions

This study provides a comprehensive evaluation of the phytochemical composition, metabolomic profile, and bioactivities of *E. clavarioides* extracts. Qualitative screening and FTIR analysis confirmed the presence of flavonoids, tannins, saponins, steroids, terpenoids, and cardiac glycosides. UHPLC-HRMS/MS profiling of the methanol extract revealed a diverse range of bioactive compounds, including phenolic acids, glycosylated metabolites, and lipophilic terpenoids, suggesting potential synergistic interactions that underpin the observed biological activities. Antioxidant assays demonstrated significant radical scavenging activity, particularly in methanol extracts, highlighting their capacity to neutralize reactive oxygen and nitrogen species. Anti-inflammatory evaluation revealed that ethanol extracts strongly inhibited nitric oxide production, while aqueous extracts elicited pro-inflammatory responses consistent with traditional wound-healing mechanisms. Anticancer assays showed that methanol and ethanol extracts exerted potent antiproliferative effects on prostate, gastric, and uterine cancer cell lines, likely mediated by the combined antioxidant, anti-inflammatory, and cytotoxic actions of polyphenols and terpenoids. These findings support the traditional medicinal use of *E. clavarioides* and underscore its potential as a source of multi-target natural therapeutic agents, warranting further bioactivity-guided and mechanistic studies.

## Figures and Tables

**Table 1:** Extraction yield of *E. clavarioides* extracts.

Extract Solvent	Yield (%)
**Aqueous**	14.5
**Ethanol**	2.8
**Methanol**	1.3
**Hexane**	0.4

**Table 2:** Qualitative phytochemical analysis of *Euphorbia clavarioides* Boiss.

S/NO	Bioactive Compound	Activity
**1**	Tannins	+
**2**	Phlobatannins	+
**3**	Saponins	+
**4**	Flavonoids	++
**5**	Alkaloids	+
**6**	Steroids	+
**7**	Terpenoids	+
	Cardiac glycosides	+

The (++) sign means strongly positive; (+) means positive and (−) means negative.

**Table 3:** FTIR interpretation of the functional group of aqueous extracts of *E. clavarioides* Boiss. aqueous extracts.

SPEC. NO	Wave Number cm^−1^ (Test Samples)	Wave Number cm^−1^ [19]	Functional Group	Phyto Compounds Identified
**1**	2990.66	3500–2400	Carboxylic acid, O–H stretch	Carboxylic acid
**2**	1738.68	1820–1670	C=O stretch	Carboxyl group
**3**	1366.02	1410–1310	O–H stretch, alcoholic group	Phenol
**4**	1217.22	1360–1210	C–N stretch	Amine
**5**	527.37	730–500	C–Cl	Alkyl halides

**Table 4:** Key metabolites in negative ion mode.

No	Tentative Identification	Molecular Formula	Calculated Mass [M–H]^−^	Observed *m*/*z*	Δ (Ppm)	Rt (Min)	Conf. Level	Peak Height Intensity
**1**	Putative sugar-derived metabolite	-	-	323.09819	-	2.31	3	26,930
**2**	Pseudolaroside B	C_14_H_18_O_9_	329.08776	329.08829	1.61	3.56	2	218,173
**3**	Cinnamtannin A2	C_60_H_50_O_24_	576.12723	576.12775	0.90	3.68	2	43,102
**4**	(−)-Medicarpin	C_16_H_14_O_4_	269.08193	269.08210	0.63	4.44	2	5134

**Table 5:** Key metabolites in positive ion mode.

No.	Tentative Identification	Molecular Formula	Calculated Mass [M + H]^+^	Observed *m*/*z*	Δ (Ppm)	Rt (Min)	Conf. Level	Peak Height Intensity
**1**	Putative small terpenoid	-	-	310.1290	-	3.16	3	32,361
**2**	Zerumbone	C_15_H_22_O	219.1749	219.1746	−1.28	7.27	2	31,460
**3**	Sclareol (isomer)	C_20_H_36_O_2_	309.2788	309.2787	−0.35	12.09	2	55,568

**Table 6:** Shows the DPPH and NO Scavenging activity of *E. clavarioides* Boiss extracts.

Sample	DPPH (IC_50_ Value µg/mL)	NO (IC_50_ Value µg/mL)
**Aqueous**	1952.50 ± 4.85 ^d^	2668.89 ± 5.98 ^b^
**Ethanol**	1913.64 ± 3.78 ^c^	1913.64 ± 3.78 ^c^
**Methanol**	755.71 ± 7.26 ^b^	755.71 ± 7.26 ^b^
**Ascorbic Acid (Control)**	373.33 ± 2.82 ^a^	373.33 ± 2.82 ^a^

Data are expressed as mean ± standard deviation. Different letters (^a–d^) in the same column indicate significant differences among samples (*p* < 0.05), where n = 3.

## Data Availability

All data generated or analyzed during this study are included in this published article.
